# Systematic Genome-wide Screening and Prediction of microRNAs in EBOV During the 2014 Ebolavirus Outbreak

**DOI:** 10.1038/srep09912

**Published:** 2015-05-26

**Authors:** Yue Teng, Yuzhuo Wang, Xianglilan Zhang, Wenli Liu, Hang Fan, Hongwu Yao, Baihan Lin, Ping Zhu, Wenjun Yuan, Yigang Tong, Wuchun Cao

**Affiliations:** 1 The State Key Laboratory of Pathogen and Biosecurity, Beijing Institute of Microbiology and Epidemiology, Beijing, China; 2 Department of Physiology and Key Laboratory of Fertility Preservation and Maintenance of Ministry of Education, Ningxia Medical University, Yinchuan 750004, PR China; 3 Computational Neuroscience Program, Department of Psychology, Physics, and Computer Science and Engineering, University of Washington, Seattle, WA 98195, USA

## Abstract

Recently, several thousand people have been killed by the Ebolavirus disease (EVD) in West Africa, yet no current antiviral medications and treatments are available. Systematic investigation of ebolavirus whole genomes during the 2014 outbreak may shed light on the underlying mechanisms of EVD development. Here, using the genome-wide screening in ebolavirus genome sequences, we predicted four putative viral microRNA precursors (pre-miRNAs) and seven putative mature microRNAs (miRNAs). Combing bioinformatics analysis and prediction of the potential ebolavirus miRNA target genes, we suggest that two ebolavirus coding possible miRNAs may be silence and down-regulate the target genes NFKBIE and RIPK1, which are the central mediator of the pathways related with host cell defense mechanism. Additionally, the ebolavirus exploits the miRNAs to inhibit the NF-kB and TNF factors to evade the host defense mechanisms that limit replication by killing infected cells, or to conversely trigger apoptosis as a mechanism to increase virus spreading. This is the first study to use the genome-wide scanning to predict microRNAs in the 2014 outbreak EVD and then to apply systematic bioinformatics to analyze their target genes. We revealed a potential mechanism of miRNAs in ebolavirus infection and possible therapeutic targets for Ebola viral infection treatment.

The genus Ebolavirus belongs to the family Filoviridae and order Mononegavirales, where the members of this genus are called ebolaviruses^1^. The Ebolavirus (EBOV, formerly designated “Zaire ebolavirus”) is one of the five known Ebolaviruses. EBOV cause Ebolavirus disease (EVD) in humans and other mammals. EVD is a type of hemorrhagic fever having a high case fatality rate^2^. From March 2014 to October 2014, the Ebola outbreak in West Africa has sickened 8,399 people, killing 4,033 of them^3^. This is the largest EBOV outbreak in the history^4^. Each month, several thousand people from affected areas travel to the North America, and even more travellers enter and leave Europe, other parts of Africa, and Asia^5^. Such air travel situations increase the possibility of EVD transmission. The United States and France have reported that several patients tested positive for Ebola^3^,^4^,^5^,^6^. Moreover, no current antiviral medications are available for EBOV^7^, and no effective EVD treatment exists at this time^8^.

MicroRNAs (miRNAs) are small regulatory non-coding RNAs, ranging from 19 to 24 nucleotides, that post-transcriptionally regulate target gene expression by inhibiting the translation of mRNA transcripts or cleaving them^9^,^10^,^11^,^12^. While encoded not only by cellular genomes but also by viral genomes^13^,^14^,^15^, miRNAs play a vital role in numerous cellular processes, including cell metabolism, viral infection, and antiviral immune response^16^,^17^. Viral miRNAs are mostly identified by traditional cloning from virus-infected cells^18^,^19^,^20^. Computational prediction and hybridization analysis are also applied to viral miRNA identification^21^,^22^. For known viral miRNAs, the majority of them are encoded by DNA viruses while only a few derived from RNA viruses^23^,^24^. To date, the RNA virus-encoded miRNAs have been identified in a few retroviruses including bovine leukemia vius (BLV)^25^,^26^, human immunodeficiency virus (HIV)^25^, West Nile virus (WNV)^27^, Dengue virus^28^ and hepatitis A virus (HAV)^23^. Recently, Liang *et al.*^24^ have identified two EBOV miRNAs, which means that EBOV can encode functional miRNAs.

Unlike the method used by Liang *et al.*^24^, based on bioinformatics whole genome-wide scanning and screening, we predicted the potential mature 2014 EBOV miRNAs, their target genes, and related signaling pathways. To the best of our knowledge, this is the first paper to systemic genome-wide analyze and predict the potential non-coding RNAs in the 2014 outbreak EBOVs along with their target genes. The results show several target genes regulated by the possible miRNAs may have important functions in human immune and antiviral response systems. Our research helps to further assess the roles of the 2014 outbreak EBOV miRNAs and their potential targets during viral infection and virus-host interactions, and thus to speed up the process of effective EVD treatment development.

## Results

### Alignment of EBOV whole genomes and prediction of mature EBOV miRNAs

**Figure 1:**
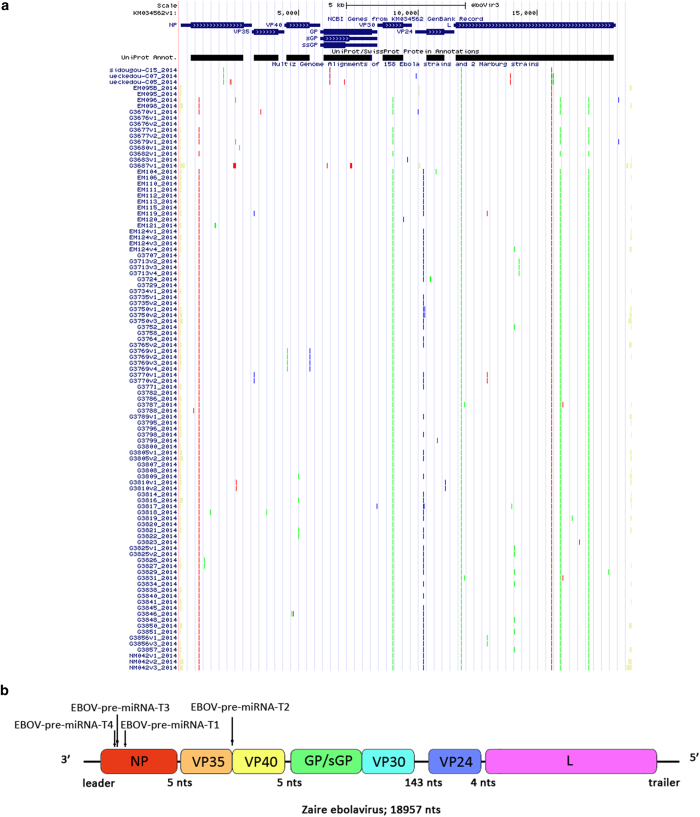
The 102 complete genome sequences alignment of the 2014 outbreak EBOV. (**A**) Full alignment map of all 102 EBOV complete genomes. (**B**) Locations and putative target sites of the four real miRNA precursor candidates.

**Figure 2:**
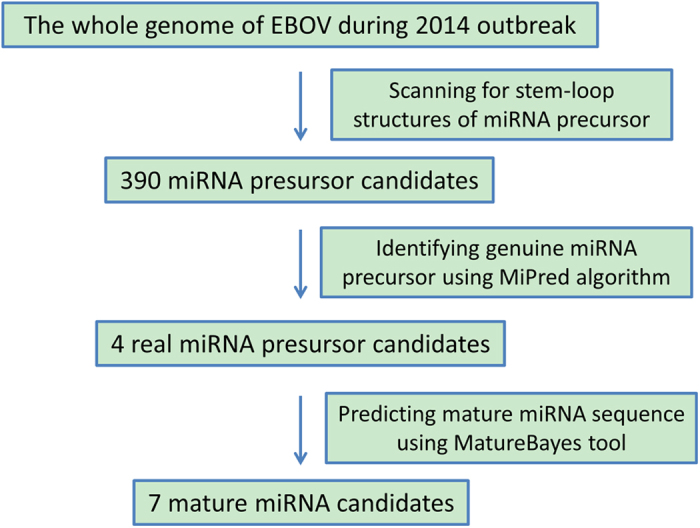
Workflow of the 2014 outbreak EBOV-encoded miRNA prediction. The MiPred algorithm was used to identify genuine pre-miRNAs, and the MatureBayes tool was used to predict the mature miRNA sequences.

As shown in Fig. 1A, we first aligned the complete genome sequences of the 102 human EBOV strains acquired from the 2014 outbreak^29^. The mutation sites were all highlighted in different colors^30^. Four real microRNA precursor candidates, with the names of EBOV-pre-miRNA-T1, EBOV-pre-miRNA-T2, EBOV-pre-miRNA-T3 and EBOV-pre-miRNA-T4, were picked out. Their sequences are listed in Table 1. EBOV-pre-miRNA-T1 is 136-base long, EBOV-pre-miRNA-T2 is 80-base long, EBOV-pre-miRNA-T3 is 99-base long, and EBOV-pre-miRNA-T4 is 99-base long. Their locations were marked in Fig. 1B. The start position of EBOV-pre-miRNA-T1 is 653, while its end position is 788. EBOV-pre-miRNA-T2 is located between the position of 4373 and of 4452. EBOV-pre-miRNA-T3 is at the range between 488 and 586. EBOV-pre-miRNA-T4 is located from the position of 479 to 568. Seven different mature EBOV miRNA candidates were predicted using the four EBOV miRNA precursor candidates. The sequences of the eight mature EBOV miRNAs are listed in Table 2, where EBV-miR-T3-5p has the same sequence with EBV-miR-T4-5p. Their lengths are all 22-base long. By aligning and blasting the seven miRNAs, including EBV-miR-T1-5p, EBV-miR-T1-3p, EBV-miR-T2-5p, EBV-miR-T2-3p, EBV-miR-T3-5p, EBV-miR-T3-3p, EBV-miR-T4-5p and EBV-miR-T4-3p, to other species of viral or host miRNAs, only EBV-miR-T3-5p has some similar miRNAs. We analyzed the conservation between EBV-miR-T3-5p and its similar miRNAs, which is described in the Table 3. A flowchart describing the computational prediction of the putative miRNAs is shown in Fig. 2.

### Structures of potential EBOV pre-miRNAs and mature miRNAs

**Figure 3:**
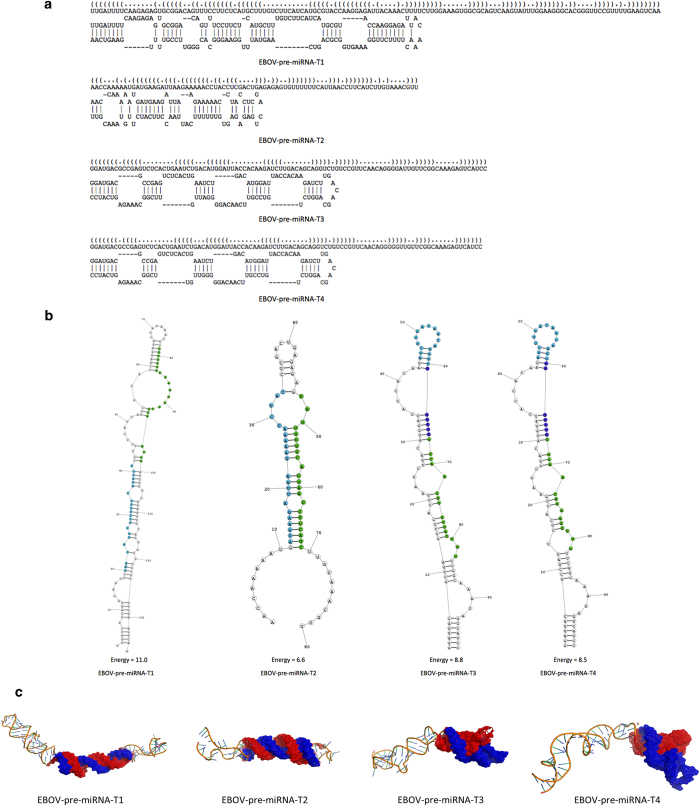
Predicted hairpin structures of potential EBOV pre-miRNAs. (**A**) The primary structures of the four EBOV pre-miRNAs. (**B**) The secondary structures of the four EBOV pre-miRNAs. (**C**) The tertiary structures of the four EBOV pre-miRNAs.

In order to achieve more accurate prediction, we predicted the primary, secondary, and tertiary structure of the four potential EBOV pre-miRNAs^31^,^32^. RNAfold was used to predict consensus secondary structures of the pre-miRNAs. Attempts to fold these pre-miRNAs, along with their flanking sequences, into the expected hairpin structures were successful in candidates EBOV-pre-miRNAs-T1, EBOV-pre-miRNAs-T2, EBOV-pre-miRNAs-T3 and EBOV-pre-miRNAs-T4, while other pre-miRNAs failed to fold into any form of stable RNA structures because that they could only give rise to stem-loops that were too short to be authentic pre-miRNA stem-loops. These findings led us to believe that EBOV-pre-miRNAs-T1, EBOV-pre-miRNAs-T2, EBOV-pre-miRNAs-T3 and EBOV-pre-miRNAs-T4 are indeed authentic EBOV pre-miRNAs. The optimal secondary structures in dot-bracket notation with a minimum free energy of −39.4 kcal/mol of EBOV-pre-miRNA-T1, −18.8 kcal/mol of EBOV-pre-miRNA-T2, −33.51 kcal/mol of EBOV-pre-miRNA-T3 and −31.91 kcal/mol of EBOV-pre-miRNA-T4 are given in Fig. 3A,B shows the predicted hairpin structures of confirmed EBOV pre-miRNAs. Hairpins longer than 100 nt were truncated; these hairpins are indicated by a double slash preceding the stem. Furthermore, the 3D structures of the potential EBOV pre-miRNAs were computationally predicted. Even though EBOV-pre-miRNA-T3 and EBOV-pre-miRNA-T4 have similar 2D structures^31^, they own different 3D structures (Fig. 3C). The positions of possible mature miRNAs were colored in second and tertiary structures (Figs. 3B,C)^32^. For EBOV-pre-miRNAs-T1, EBOV-pre-miRNAs-T2, EBOV-pre-miRNAs-T3 and EBOV-pre-miRNAs-T4 alternative predictions, which are likely to represent the authentic stem-loop structures recognized and cleaved by Drosha are shown underneath the minimal energy structures.

### Bioinformatics analysis of mature EBOV miRNAs and prediction of their target genes

Based on the sequences of the seven EBOV miRNAs, EBV-miR-T1-5p, EBV-miR-T1-3p, EBV-miR-T2-5p, EBV-miR-T2-3p, EBV-miR-T3-5p, EBV-miR-T3-3p, EBV-miR-T4-5p and EBV-miR-T4-3p, their target genes were searched by TargetScan^33^ and the other miRNA regulation database (the integration database of miRecords, TarBase, starbase, and miR2Disease)^34^,^35^,^36^,^37^. All these potential mature miRNAs have total 138 possible target genes in human genome (Table 4). Interestingly, the genes P11, EFNA3, FKRP, AMBRA1, HEPACAM, HCCA2, LPHN1 and PHF21B were all targeted by at least two potential mature EBOV miRNAs.

**Figure 4:**
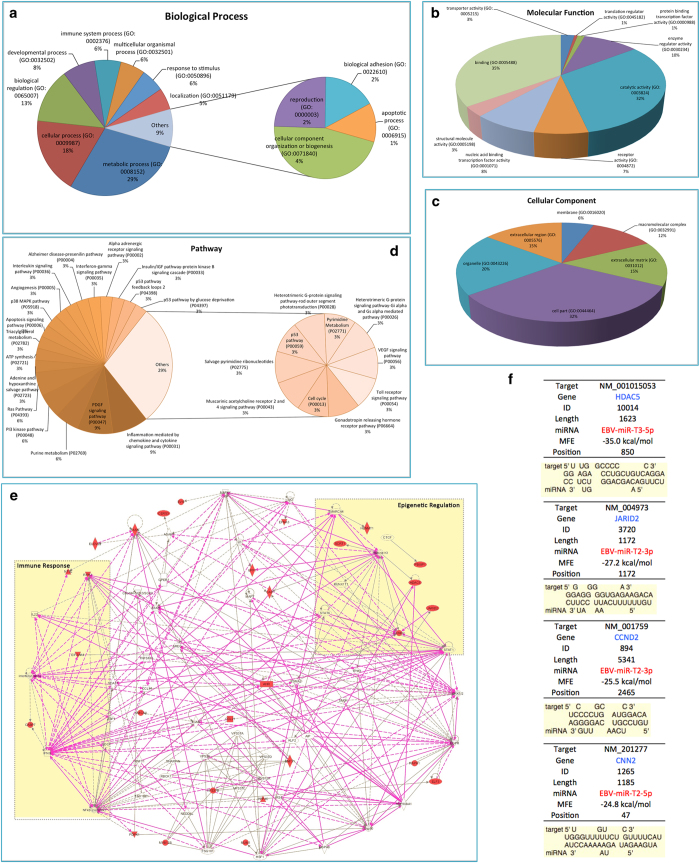
Bioinformatic analysis of target gene prediction and related signaling pathways of the potential mature miRNAs in EBOV. The predicted target gene of potential mature EBOV miRNAs were classified by the GO and DAVID databases based on biological process (**A**), molecular function (**B**) and cellular compartment (**C**). (**D**) The pathway enrichment analysis of candidate genes. Top enriched pathways are listed (*p value* < 0.01). (**E**) The gene regulation network analysis of the potential target genes. (**F**) The detail information of target genes of miRNA EBV-miR-T3-5p, EBV-miR-T2-3p, EBV-miR-T2-3p and EBV-miR-T2-5p.

Using the GO and DAVID databases^38^,^39^, identified proteins were clustered into groups based on their biological processes, molecular function, and cellular compartment. Such clustering allowed us to determine how the differentially expressed proteins were distributed according to biological process (Fig. 4A), molecular function (Fig. 4B) and cellular compartment (Fig. 4C). Clusters with the biological process included proteins that are involved in immune system process (GO:0002376), multicellular organismal process (GO:0032501) and response to stimulus (GO:0050896), which are important for human antiviral response (Fig. 4A). This analysis grouped proteins into nine molecular functional classes (Fig. 4B), which were associated with transcription regulation, such as transporter activity (GO:0005215), translation regulator activity (GO:0045182), protein binding transcription factor activity (GO:0000988) and enzyme regulator activity (GO:0030234). Protein clusters were then further categorized into six subcellular distributions according to the cellular compartment (Fig. 4C). Fig. 4D shows the pathway enrichment analysis of the target genes. Kyoto Encyclopedia of Genes and Genomes (KEGG) pathway enrichment analysis was performed using the DAVID bioinformatics tool^40^,^41^. The top enriched pathways are listed here (p-value < 0.01). Fig. 4D clearly shows that these proteins are functionally closely related, and are involved in multiple pathways. Inflammation are mediated by chemokine and cytokine signaling pathway (P00031), PDGF signaling pathway (P00047), Purine metabolism (P02769), PI3 kinase pathway (P00048) and Ras Pathway (P04393), which are important in human immune response to virus infection.

The gene regulation network (GRN) of the target genes^42^,^43^ is shown in the Fig. 4E. We find that the possible target genes, HDAC5 and JARID2^44^, are important epigenetic factors in transcription regulation, which have strong interaction with the transcription regulator in human cells, such as Histone h3, STAT1, CTCF, CCND2 and CTCF. It is illustrated that the EBOV down-regulated the epigenetic factor to large-scale epigenetic alterations in the host gene expressions^45^, which will damage the normal molecular regulation in immune signaling and thus disorder human immune system to block the anti-viral response. The target proteins, NFKBIE and RIPK, are the key co-regulator with IL22 and IFNG or the part of complex of NFκB in human immune system^46^,^47^. It also suggests that the EBOV affects the signaling pathway of immune system by the non-coding RNA to inhibit the infection response. Further network analysis was conducted to see the potential biological pathways and processes in which these differentially expressed proteins were involved. In addition, Fig. 4F illustrates the detail information of target genes which play key roles in important immune and epigenetic responses.

### Signaling pathway analysis of the target genes

**Figure 5:**
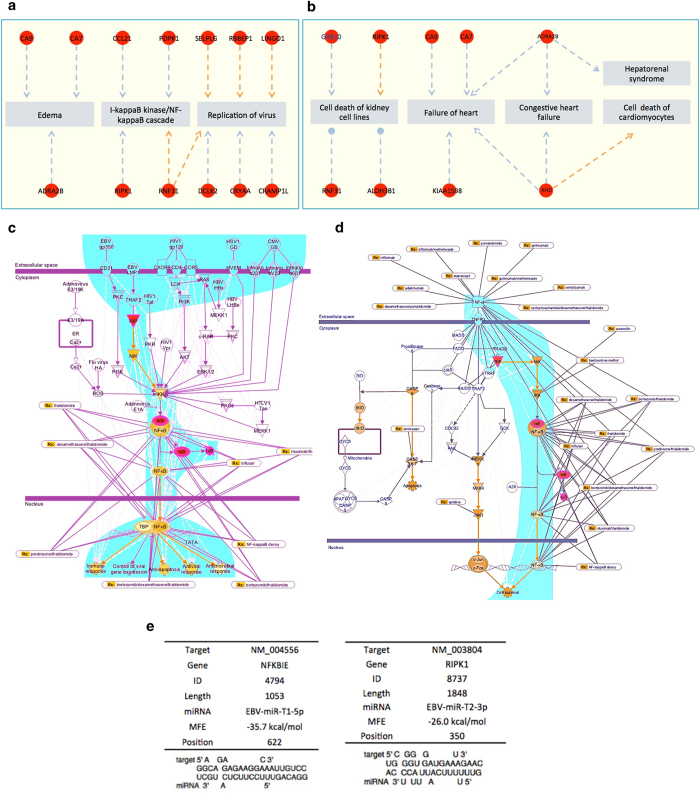
Signaling pathway analysis of the potential mature EBOV miRNA target genes. (**A**) The associated genes of Edema, □-kappaB kinase/ NF-kappaB cascade and replication of virus. (**B**) The associated genes of cell death of kidney cell lines, failure of heart, congestive heart failure, hepatorenal syndrome and cell death of cardiomyocytes. (**B**) TNF signaling pathway. (**C**) NF-κB signaling pathway. (**D**) Detail information of target gene NFKBIE. (**E**) Detail information of target gene RIPK1.

According to our gene regulation network analysis, NFKBIE and RIPK1 are two target genes of possible mature miRNAs, EBOV-miR-T1-5p and EBOV-miR-T2-3p, which plays important roles in immune system. Fig. 5A,B show the cytotoxin-associated target genes. NFKBIE^48^ and RIPK^49^ genes are both involved in the NF-κB and TNF signaling pathways (Fig. 5C,D). NFKBIE encodes IκB epsilon (IκBε), a member of the IκB family. Its binding to NF-κB inhibits the nuclear translocation of NF-κB^50^. RIPK1, Receptor-interacting protein 1, is a key effector molecule in the TNFα-induced activation of the transcription factor NF-κB^51^. The signaling pathway analysis shows that the TNF-a–mediated NF-kB signaling pathway plays a key role in inflammatory response (Fig. 5C,D). Through analyzing the molecular mechanism in the NF-kB and TNF signaling pathways, those two pathways were discovered to participate in the host immune response, thus they are also attractive targets of viral pathogens^52^. The EBOV coding possible miRNAs, EBOV-miR-T1-5p and EBOV-miR-T2-3p, may be silence and down-regulate the NFKBIE and RIPK1, which are the central mediator of those pathways. In addition, the EBOV exploits the non-coding RNA to inhibit the NF-kB and TNF factor to evade the host defense mechanisms that limit replication by killing infected cells, or to conversely trigger apoptosis as a mechanism to increase virus spreading. NF-kB is activated by multiple families of viruses, including HIV-1, HTLV-1, hepatitis B virus (HBV), hepatitis C virus (HCV), EBV, and influenza virus^53^. This activation may serve several functions of promoting viral replication, preventing virus-induced apoptosis, and suprressing the immune response to the invading pathogen^54^. The genes NF-kB and TNF are affected by synthetic dsRNAs. It suggests that viruses that generate dsRNA replicative intermediates employ a common mechanism to enhance viral replication. The EBOV glycoprotein (GP) and the other viral proteins also induce the NF-kB and TNF signaling pathways^55^. The miRNA and target gene details of EBV-miR-T1-5p and EBV-miR-T2-3p are listed in Fig. 5E. The target gene of EBV-miR-T1-5p is named NFKBIE with ID No. 4794, and 1053-bp long. The target gene of EBV-miR-T2-3p is named RIPK1 with ID No. 8737, and 1848-bp long. Normally when a virus enters a human body, the target genes mentioned above should increase their expression levels. Based on our analysis, once EBOV enters the human body, the miRNAs EBV-miR-T1-5p and EBV-miR-T2-3p down-regulate the target genes of NFKBIE and RIPK1 to evade host innate immune responses and finally leads to multiple organ dysfunction syndrome (MODS).

### EBOV miRNA regulated the expression of target genes NFKIBIE, RIPIK1, HDAC5 and JARID2

**Figure 6:**
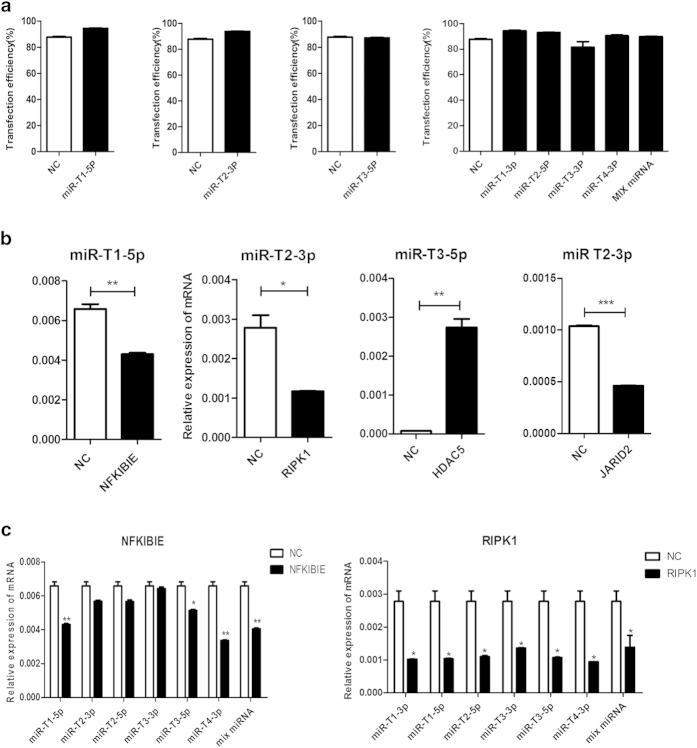
Transfection efficiency of EBOV-miRNA mimics and effect of the EBOV-miRNA mimics on the expression of target genes in Hela cells. (**A**) Transfection efficiency 24 h post-transfection in Hela cells as percentage of fluorescent dye labeled miRNA positive cells. (**B**) Relative expression levels of NFKIBIE, RIPK1, HDAC5 and JARID2, which were regulated by EBOV-miRNA mimics miR-T1-5p, miR-T2-3p and miR-T3-5p, respectively. (**C**) Relative expression levels of NFKIBIE and RIPK1, which were regulated by six EBOV-miRNA mimics and their mixed miRNA (MIX miRNA). *: P < 0.05, **: P < 0.01, ***: P < 0.001. Label “NC” means negative control.

The miRNA mimics was synthesized and transfected into Hela cells for 24 h/37 °C. The results showed that EBOV-miRNA mimics directly regulate the expression of target genes, NFKIBIE, RIPIK1, JARID2 and HDAC5, in Hela cells. Real time (RT)-PCR was used to quantify and compare the mRNA expression of target genes in the transfected Hela cells with the EBOV-miRNA mimics and the scramble mimics as negative control. The transfection efficiency of miRNA mimics, including miR-T1-3p, miR-T2-5p, miR-T3-3p, miR-T4-3p and their mixed miRNA (MIX miRNA), and the scrambled miRNA mimics were detected by fluorescence activated cell sorter (FACS), which were more than 80% (Fig. 6A). The RT-PCR analysis indicated that expression of NFKIBIE was significantly down-regulated by 1.526 times in miR-T1-5p mimics-transfected Hela cells and the expression of RIPIK1 and JARID2 were down-regulated by miR-T2-3p mimics with 1.947 times and 2.25 times separately (Fig. 6B). Interestingly, in the transfected Hela cells with miR-T3-5p mimics, the expression of HDAC5 is increasing compared with the transfected Hela cells with scrambled miRNA mimics (Fig. 6B). We also found that the mRNA expression of NFKIBIE were inhibited by miR-T1-3p, miR-T3-5p, miR-T4-3p and MIX miRNA mimics, while all EBOV-miRNA mimics significantly can block the expression of RIPK1 (Fig. 6C). Such results illustrated that these miRNA may have important functions in human immune and antiviral response systems.

## Conclusion

From March 2014 to October 2014, the largest EBOV outbreak in the history has killed several thousand people in West Africa, yet no current specific treatment is validated for EBOV. miRNAs has been demonstrated to circulate in a highly stable, cell-free form in body fluids thus circulating miRNAs can be used as non-invasive biomarkers for molecular diagnostics and prognostics^24^. Since the majority of known viral miRNAs are encoded by DNA viruses, RNA-virus-encoded miRNAs remains controversial. Recently, despite that several novel miRNAs have been experimentally identified in the West Nile virus (WNV)^28^, Dengue virus (DENV)^56^, hepatitis A virus (HAV)^23^, the viral miRNAs in numerous viruses have been identified using computational prediction followed by experimental validation^23^,^57^,^58^,^59^. This year H. Liang *et al.*^24^ have successfully identified two EBOV miRNAs, which provide new evidence that EBOV can encode functional miRNAs.

In this paper, based on the whole genome alignment of the 2014 outbreak EBOVs^29^, which is different from the analysis method of Liang *et al.*^24^, we predicted and experimentally verified seven mature EBOV miRNAs and their regulated target genes. By analyzing the signaling pathways of the target genes, we hypothesized that EBOV miRNAs, especially EBV-miR-T1-5p and EBV-miR-T2-3p, down-regulate the target genes of NF-KB and TNF expression which are involved in virus-cell interaction, immune escape, and cell apoptosis^48^,^49^. As viruses evolve under the highly selective pressures of the immune system, they acquire the capacity to target critical steps in the host cell life, hijacking vital cellular functions to promote viral pathogenesis. Many viruses have evolved mechanisms to target the NF-kB pathway to facilitate their replication, cell survival, and evasion of immune responses. In addition, some viruses use the NF-kB pathway either for its anti-apoptotic properties to evade the host defense mechanisms or to trigger apoptosis as a mechanism of virus spread. Not surprisingly, recent studies focusing on the interrelations between NF-kB and virus pathogenesis reveal that many viral products bypass signal-induced stimulation and/or receptor-proximal steps to directly interface with the IKK complex. Continuous activation of NF-kB as a consequence of viral persistence in some instances leads to oncogenic transformation, which is an area of increasing scientific study and fascination.

It is noteworthy that this is the first paper to describe a strategy that the EBOV non-coding RNA may be evolved to modulate the NF-kB and TNF signaling pathways, to enhance viral replication, host cell survival, and evasion of immune responses. According to our prediction results, the EBOV-miRNA mimics of miR-T1-5p can significantly inhibit the expression of NFKIBIE, while the miR-T2-3p mimics can block the expression of RIPK1 significantly.

Such results illustrated that these miRNA may have important functions in human immune and antiviral response systems. Ongoing research will undoubtedly continue to reveal other novel interactions between viruses and the NF-kB pathway that will permit a more precise molecular dissection of this parasitic fatal attraction. Considering that the target genes regulated by our predicted miRNAs play key roles in human immune response, we highlight our prediction work raises the hope of quickly finding an effective EBOV disease treatment.

## Materials and Methods

### EBOV whole genome sequencing

To show the genome-wide nucleotide and amino acid signatures, we retrieved full genome sequences (as of October 22, 2014) from the genome browser at NCBI Database. One-hundread-and-two human EBOV genomes were derived from the 2014 outbreak^29^,^30^.

### Multiple genome-wide alignments

The multiple sequence alignment tools ClustalW and MUSCLE were applied for the alignment of the EBOV genomes. The alignments were then analyzed to identify the characteristic sites as potential signatures to distinguish different virus genomes and proteins^30^.

### Bioinformatics prediction of the miRNAs

A flowchart describing the computational prediction of the putative miRNAs is shown in Fig. 2. Briefly, the viral genome was scanned for stem-loop structures of miRNA precursor (pre-miRNA) using VMir^60^ (http://www.hpi-hamburg.de/research/ departments-and-research-groups/antiviral-defense-mechanism/software-down- load.html), a computational analyzer program for the prediction of putative pre-miRNAs. The complete genome sequences of 138 different human EBOV strains were acquired from the PubMed, where 102 human EBOV strains belong to 2014 EBOV outbreak. VMir predictions were carried out using the default parameters. The putative pre-miRNAs that satisfied the filter parameters of a VMir score ≥150 and a window count ≥35 were selected for further assessment. Then, the source code of MiPred^61^ was used to distinguish real and pseudo miRNA precursors from the obtained sequences with a prediction confidence equal to or greater than 70%. Subsequently, mature miRNA sequences were predicted from the pre-miRNA stem-loops. Then we used the MatureBayes tool^62^ to extend the prediction coverage of the mature miRNAs. The default conditions were used for the MatureBayes tool.

### Prediction of Novel Viral miRNA Targets

Human target genes of novel EBOV miRNAs were predicted using TargetScan^33^ custom miRNA prediction methods. Putative targets within the viral genome were predicted using TargetScan Perl script^34^,^36^,^37^,^60^.

### Gene ontology (GO) analysis

GO analysis of the significant probe list was performed using PANTHER (http://www.pantherdb.org/)^38^,^39^, using text files containing the Gene ID list and accession numbers of the Illumina probe ID. All data analysis and visualization of differentially expressed genes were conducted using R 2.4.1 (www.r-project.org). In addition, the DAVID Functional Annotation Bioinformatics Microarray Analysis tools (http://david.abcc.ncifcrf.gov/) were used to study the biological function of the regulated genes.

### Kyoto Encyclopedia of Genes and Genomes (KEGG) pathway analysis

Kyoto Encyclopedia of Genes and Genomes (KEGG)^40^,^41^ is a collection of online databases dealing with genomes, enzymatic pathways, and biological chemicals. The PATHWAY database records networks of molecular interactions in the cell that includes organism-specific network maps (http://www.genome.jp/kegg/). A total of 9 pathways, involving 49 genes, were collected from KEGG.

### Constructing Gene Regulation Network

The integrated regulatory network was constructed based on systematic integration of various high-throughput datasets^42^,^43^. The target genes associated with candidate miRNAs were selected from miRNA regulation database (the integration database of miRecords, TarBase, starbase, and miR2Disease) and TFs associated with those target genes were selected from Transcriptional Regulatory Element Database (TRED). The integrated target genes-TF regulatory network was constructed by using Cytoscape software (http://cytoscape.org/), which is an open source software for visualizing complex networks and integrating these networks with any type of attribute data.

### Cell culture and transfection

Hela cells at 37 °C and 5% CO_2_ in Dulbecco’s modified Eagle’s medium (DMEM) supplemented with 10% fetal bovine serum (Hyclone, USA) . Synthetic duplex EBV-miRNA mimics, scramble oligonucleotides used as negative control (NC) (GenePharma, Shanghai, China) at a final concentration of 50 nM were introduced into Hela cells by siPORT™ NeoFX™ transfection agent (AM4511, Applied Biosystems Inc., USA). according to the manufacturer’s instructions. Cells were harvested at 24 hours after transfection.

### RNA extraction and quantitative real-time PCR analysis

The harvested cells were placed in TRIzolH Reagent (Invitrogen, Carlsbad,CA), and total RNAs were extracted according to the TRIzol manufacturer’sinstructions. Total RNA (3 μg) was primed by oilgodT and converted intocDNA using SuperScript III (Invitrogen). The SYBR green-based real-time PCR was performed in Light Cycle 2.0 System(Roche) and Relative quantification of mRNA was measured using 2-ΔΔCt method which normalized to GAPDH. Each PCR assay was performed in a final volume of 20 ul, containing 2 ul of DNA template, 25 pmol of each type-specific primer set, 2X Maxima SYBR Green (Thermo Scientific), Reactions were incubated at 50 °C for2 minutes, followed by PCR amplification under the following conditions: 95 °C for10 minutes, 95 °C for 30 seconds, 57 °C for 30 seconds, 72 °C for 30 minute for a total of 40 cycles, Following the PCR step, a melt curve analysis was performed by increasing the temperature from 65 °C to 95 °C at increments of 0.1 °C/s for each fluorescence reading. The primer sequences for qPCR analysis were as follows: GAPDH, forward 5′-AGAAGGCTGGGGCTCATTTG-3′ and reverse 5′- AGGGGCCATCCACAGTCTTC-5′; NFKBIE, forward 5′- TGCCAACAGATGGCCCATAC-3′ and reverse 5′- TGTTCTTTTCACTAGAGGCACCA-3′; RIPK1, forward 5′-TGGGCGTCATCATGAGGAAG-3′ and reverse 5′-CGCCTTTTCCATGTAAGTAGCA-3′ ; MAPKAP3, forward 5′- ATGAGAACATGCACCATGGCAAGC-3′ and reverse 5′- GGGCAATGTTATGGCTGTGCAGAA -3′; HDAC5, forward 5′- TTCTTTGGACCAGAGTTCCC-3′ and reverse 5′- GTTGGGTTCAGAGGCTGTTT-3′; JARID2, forward 5′- GAGCATGTGTTTCAGCAAGG-3′ and reverse 5′- CTTCTCTTCCACTAGCCTCCAG-3′

### Flow cytometry

Green fluorescence after fluorescent dye labeled miRNA delivery into Hela cells were assessed in an Accuri C6 BD Biosciences flow cytometer, excited by an argon 480 nm laser and detected by use of a 520 nm optical filter. The mean fluorescence intensity (MFI) data were collected from these cell populations. All data was analyzed with a minimum of 15000 events setting the respective negative control to less than 1% in BD CFlow software. Post-transfection in Hela cells as percentage of fluorescent dye labeled miRNA positive cells.

## Additional Information

**How to cite this article**: Teng, Y. *et al*. Systematic Genome-wide Screening and Prediction of microRNAs in EBOV During the 2014 Ebolavirus Outbreak. *Sci. Rep.* **5**, 9912; doi: 10.1038/srep09912 (2015).

## Figures and Tables

**Table 1:** The predicted pre-miRNAs in EBOV.

**Pre-miRNA**	**Strand**	**Start Position**	**End Position**	**Length**	**Sequence (5′-3′)**
EBOV-pre-miRNA-T1	Plus	653	788	136	UUGAUUUUCAAGAGAGUGCGGACAGUUUCCUUCUCAUGCUUUGUCUUCAUCAUGCGUACCAAGGAGAUUACAAACUUUUCUUGGAAAGUGGCGCAGUCAAGUAUUUGGAAGGGCACGGGUUCCGUUUUGAAGUCAA
EBOV-pre-miRNA-T2	Plus	4373	4452	80	AACCAAAAAUGAUGAAGAUUAAGAAAAACCUACCUCGACUGAGAGAGUGUUUUUUCAUUAACCUUCAUCUUGUAAACGUU
EBOV-pre-miRNA-T3	Plus	488	586	99	GGAUGACGCCGAGUCUCACUGAAUCUGACAUGGAUUACCACAAGAUCUUGACAGCAGGUCUGUCCGUUCAACAGGGGAUUGUUCGGCAAAGAGUCAUCC
EBOV-pre-miRNA-T4	Plus	479	568	99	GGAUGACGCCGAGUCUCACUGAAUCUGACAUGGAUUACCACAAGAUCUUGACAGCAGGUCUGUCCGUUCAACAGGGGGUUGUUCGGCAAAGAGUCAUCC

**Table 2:**
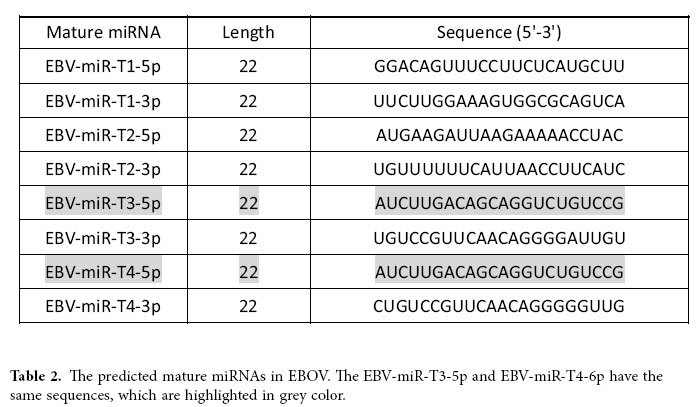
The predicted mature miRNAs in EBOV. The EBV-miR-T3-5p and EBV-miR-T4-6p have the same sequences, which are highlighted in grey color.

**Table 3:**
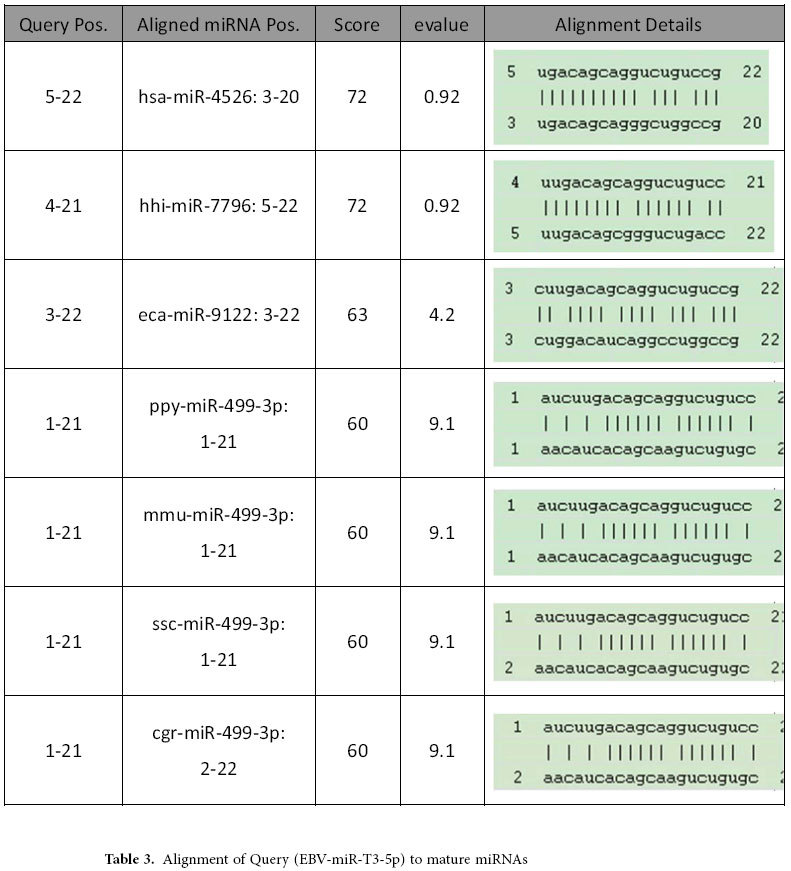
Alignment of Query (EBV-miR-T3-5p) to mature miRNAs

**Table 4:** The list of possible target genes.

**Gene Name**	**Gene ID**	**Human Symbol**
ABCC13	150000	ABCC13 (ATP-binding cassette, sub-family C (CFTR/MRP), member 13, pseudogene)
ADNP2	22850	ADNP2 (ADNP homeobox 2)
ADRA2B	151	ADRA2B (adrenoceptor alpha 2B)
ADTRP	84830	ADTRP (androgen-dependent TFPI-regulating protein)
AHR	196	AHR (aryl hydrocarbon receptor)
ALDH3B1	221	ALDH3B1 (aldehyde dehydrogenase 3 family, member B1)
AMBRA1	55626	AMBRA1 (autophagy/beclin-1 regulator 1)
AMICA1	120425	AMICA1 (adhesion molecule, interacts with CXADR antigen 1)
ARFIP1	27236	ARFIP1 (ADP-ribosylation factor interacting protein 1)
ARHGAP27	201176	ARHGAP27 (Rho GTPase activating protein 27)
ATXN10	25814	ATXN10 (ataxin 10)
BEST4	266675	BEST4 (bestrophin 4)
C2orf27A	29798	C2orf27A (chromosome 2 open reading frame 27A)
CA7	766	CA7 (carbonic anhydrase VII)
CA9	768	CA9 (carbonic anhydrase IX)
CABP7	164633	CABP7 (calcium binding protein 7)
CBX4	8535	CBX4 (chromobox homolog 4)
CCL21	6366	CCL21 (chemokine (C-C motif) ligand 21)
CCND2	894	CCND2 (cyclin D2)
CEP55	55165	CEP55 (centrosomal protein 55kDa)
CERS3	204219	CERS3 (ceramide synthase 3)
CNN2	1265	CNN2 (calponin 2)
CPSF7	79869	CPSF7 (cleavage and polyadenylation specific factor 7, 59kDa)
CRAMP1L	57585	CRAMP1L (Crm, cramped-like (Drosophila))
CRYAA	1409	CRYAA (crystallin, alpha A)
CTDSPL2	51496	CTDSPL2 (CTD (carboxy-terminal domain, RNA polymerase II, polypeptide A) small phosphatase like 2)
CYB561	1534	CYB561 (cytochrome b561)
DCLK2	166614	DCLK2 (doublecortin-like kinase 2)
DEAF1	10522	DEAF1 (DEAF1 transcription factor)
DMWD	1762	DMWD (dystrophia myotonica, WD repeat containing)
EFNA3	1944	EFNA3 (ephrin-A3)
EGFLAM	133584	EGFLAM (EGF-like, fibronectin type III and laminin G domains)
EIF2AK4	440275	EIF2AK4 (eukaryotic translation initiation factor 2 alpha kinase 4)
ELF3	1999	ELF3 (E74-like factor 3 (ets domain transcription factor, epithelial-specific ))
ELFN2	114794	ELFN2 (extracellular leucine-rich repeat and fibronectin type III domain containing 2)
ELOVL2	54898	ELOVL2 (ELOVL fatty acid elongase 2)
ENDOU	8909	ENDOU (endonuclease, polyU-specific)
EVI5	7813	EVI5 (ecotropic viral integration site 5)
EXOSC6	118460	EXOSC6 (exosome component 6)
FAM20C	56975	FAM20C (family with sequence similarity 20, member C)
FAM213B	127281	FAM213B (family with sequence similarity 213, member B)
FASN	2194	FASN (fatty acid synthase)
FBXL7	23194	FBXL7 (F-box and leucine-rich repeat protein 7)
FKRP	79147	FKRP (fukutin related protein)
FLJ46026	400627	FLJ46026 (FLJ46026 protein)
FOXK1	221937	FOXK1 (forkhead box K1)
G2E3	55632	G2E3 (G2/M-phase specific E3 ubiquitin protein ligase)
GCOM1	145781	GCOM1 (GRINL1A complex locus 1)
GMEB2	26205	GMEB2 (glucocorticoid modulatory element binding protein 2)
GNAT1	2779	GNAT1 (guanine nucleotide binding protein (G protein), alpha transducing activity polypeptide 1)
GPR64	10149	GPR64 (G protein-coupled receptor 64)
GRB10	2887	GRB10 (growth factor receptor-bound protein 10)
GTPBP1	9567	GTPBP1 (GTP binding protein 1)
HDAC5	10014	HDAC5 (histone deacetylase 5)
HENMT1	113802	HENMT1 (HEN1 methyltransferase homolog 1 (Arabidopsis))
HEPACAM	220296	HEPACAM (hepatic and glial cell adhesion molecule)
HES3	390992	HES3 (hes family bHLH transcription factor 3)
JARID2	3720	JARID2 (jumonji, AT rich interactive domain 2)
JRK	8629	JRK (Jrk homolog (mouse))
KIAA1244	57221	KIAA1244 (KIAA1244)
KIAA1598	57698	KIAA1598 (KIAA1598)
LINGO1	84894	LINGO1 (leucine rich repeat and Ig domain containing 1)
LOC284395	284395	LOC284395 (uncharacterized LOC284395)
LOC388022	388022	LOC388022 (uncharacterized LOC388022)
LOC388942	388942	LOC388942 (uncharacterized LOC388942)
LOC401134	401134	LOC401134 (uncharacterized LOC401134)
LOC440600	440600	LOC440600 (uncharacterized LOC440600)
LOC441455	441455	LOC441455 (makorin ring finger protein 1 pseudogene)
LPGAT1	9926	LPGAT1 (lysophosphatidylglycerol acyltransferase 1)
LPHN1	22859	LPHN1 (latrophilin 1)
LRRC27	80313	LRRC27 (leucine rich repeat containing 27)
MAPKAPK3	7867	MAPKAPK3 (mitogen-activated protein kinase-activated protein kinase 3)
MBIP	51562	MBIP (MAP3K12 binding inhibitory protein 1)
MMP25	64386	MMP25 (matrix metallopeptidase 25)
MOB2	81532	MOB2 (MOB kinase activator 2)
MOB3A	126308	MOB3A (MOB kinase activator 3A)
MOB3B	79817	MOB3B (MOB kinase activator 3B)
MRE11A	4361	MRE11A (MRE11 meiotic recombination 11 homolog A (S. cerevisiae))
MTMR11	10903	MTMR11 (myotubularin related protein 11)
MTMR6	9107	MTMR6 (myotubularin related protein 6)
MUM1	84939	MUM1 (melanoma associated antigen (mutated) 1)
MVB12B	89853	MVB12B (multivesicular body subunit 12B)
NAP1L4	4676	NAP1L4 (nucleosome assembly protein 1-like 4)
NFKBIE	4794	NFKBIE (nuclear factor of kappa light polypeptide gene enhancer in B-cells inhibitor, epsilon)
NLGN4X	57502	NLGN4X (neuroligin 4, X-linked)
NPLOC4	55666	NPLOC4 (nuclear protein localization 4 homolog (S. cerevisiae))
NT5E	4907	NT5E (5′-nucleotidase, ecto (CD73))
NUBP2	10101	NUBP2 (nucleotide binding protein 2)
PAQR4	124222	PAQR4 (progestin and adipoQ receptor family member IV)
PBXIP1	57326	PBXIP1 (pre-B-cell leukemia homeobox interacting protein 1)
PDPK1	5170	PDPK1 (3-phosphoinositide dependent protein kinase 1)
PHF20	51230	PHF20 (PHD finger protein 20)
PHF21B	112885	PHF21B (PHD finger protein 21B)
POLR2M	81488	POLR2M (polymerase (RNA) II (DNA directed) polypeptide M)
PPP4C	5531	PPP4C (protein phosphatase 4, catalytic subunit)
PRKAB2	5565	PRKAB2 (protein kinase, AMP-activated, beta 2 non-catalytic subunit)
PRKY	5616	PRKY (protein kinase, Y-linked, pseudogene)
PTPRT	11122	PTPRT (protein tyrosine phosphatase, receptor type, T)
RABEP1	9135	RABEP1 (rabaptin, RAB GTPase binding effector protein 1)
RAPH1	65059	RAPH1 (Ras association (RalGDS/AF-6) and pleckstrin homology domains 1)
RBM15B	29890	RBM15B (RNA binding motif protein 15B)
RBMS3	27303	RBMS3 (RNA binding motif, single stranded interacting protein 3)
RECQL5	9400	RECQL5 (RecQ protein-like 5)
RHBDL1	9028	RHBDL1 (rhomboid, veinlet-like 1 (Drosophila))
RIPK1	8737	RIPK1 (receptor (TNFRSF)-interacting serine-threonine kinase 1)
RNF185	91445	RNF185 (ring finger protein 185)
RNF31	55072	RNF31 (ring finger protein 31)
S100A10	6281	S100A10 (S100 calcium binding protein A10)
SAMD4B	55095	SAMD4B (sterile alpha motif domain containing 4B)
SCRT1	83482	SCRT1 (scratch family zinc finger 1)
SELPLG	6404	SELPLG (selectin P ligand)
SETD8	387893	SETD8 (SET domain containing (lysine methyltransferase) 8)
SGTA	6449	SGTA (small glutamine-rich tetratricopeptide repeat (TPR)-containing, alpha)
SLITRK1	114798	SLITRK1 (SLIT and NTRK-like family, member 1)
SOCS4	122809	SOCS4 (suppressor of cytokine signaling 4)
STC2	8614	STC2 (stanniocalcin 2)
SYNGR1	9145	SYNGR1 (synaptogyrin 1)
TBL2	26608	TBL2 (transducin (beta)-like 2)
THOC3	84321	THOC3 (THO complex 3)
TM9SF4	9777	TM9SF4 (transmembrane 9 superfamily protein member 4)
TMEM63A	9725	TMEM63A (transmembrane protein 63A)
TRIM35	23087	TRIM35 (tripartite motif containing 35)
UPP1	7378	UPP1 (uridine phosphorylase 1)
USP45	85015	USP45 (ubiquitin specific peptidase 45)
UTS2B	257313	UTS2B (urotensin 2B)
VAX2	25806	VAX2 (ventral anterior homeobox 2)
VPS37B	79720	VPS37B (vacuolar protein sorting 37 homolog B (S. cerevisiae))
WASF2	10163	WASF2 (WAS protein family, member 2)
WFIKKN2	124857	WFIKKN2 (WAP, follistatin/kazal, immunoglobulin, kunitz and netrin domain containing 2)
XDH	7498	XDH (xanthine dehydrogenase)
YY1AP1	55249	YY1AP1 (YY1 associated protein 1)
ZCCHC24	219654	ZCCHC24 (zinc finger, CCHC domain containing 24)
ZFP91	80829	ZFP91 (ZFP91 zinc finger protein)
ZMIZ2	83637	ZMIZ2 (zinc finger, MIZ-type containing 2)
ZNF444	55311	ZNF444 (zinc finger protein 444)
ZNF672	79894	ZNF672 (zinc finger protein 672)
ZNF767P	79970	ZNF767P (zinc finger family member 767, pseudogene)
ZNF804A	91752	ZNF804A (zinc finger protein 804A)
